# Visual Impairment and the Incidence of Concussions Among Youth Football Athletes

**DOI:** 10.1177/23259671261440185

**Published:** 2026-05-14

**Authors:** Karim Jamaleddine, Marina Gad El Sayed, Daniel Novak, Aubree Goodman, Jackson Helms, John Schlechter

**Affiliations:** † School of Medicine, University of California, Riverside, Riverside, California, USA; ‡ Department of Orthopedic Surgery, Riverside University Health System, Moreno Valley, California, USA; Investigation performed at University of California, Riverside, School of Medicine, Riverside, California, USA

**Keywords:** concussion, youth, football, visual impairment

## Abstract

**Background:**

Concussions are a prevalent concern in contact sports, particularly among youth American football players. While extensive research has examined concussion mechanics, the relationship between visual impairment and concussion risk remains understudied.

**Purpose:**

To investigate the incidence of concussions among visually impaired versus non–visually impaired youth football players to inform clinical practice and preventive measures.

**Study Design:**

Cohort study; Level of evidence, 3.

**Methods:**

A retrospective cohort study was performed using the TriNetX US Collaborative Network. Pediatric athletes aged 5 to 17 years with documented football participation were identified using International Classification of Diseases, Tenth Revision, codes. Visual impairment (H46-H47, H52-H54) was required to precede the index football encounter by ≥1 month. Concussions (S06.0) and concussion with loss of consciousness (LOC) (S06.0X1-S06.0X9) were assessed within 15- and 30-day windows.

**Results:**

After matching, 3674 athletes with visual impairment were compared with 3674 controls. Overall concussion incidence did not differ at 15 days (1.65% vs 1.47%; risk ratio [RR], 1.12; 95% CI, 0.75-1.68) or 30 days (1.79% vs 1.36%; RR, 1.09; 95% CI, 0.74-1.59). However, concussion with LOC was significantly more frequent among visually impaired athletes at 15 days (RR, 2.12; 95% CI, 1.19-3.76) and 30 days (RR, 2.11; 95% CI, 1.21-3.69). In the refractive impairment subgroup, overall concussion remained nonsignificant, while concussion with LOC was significantly increased at 15 days (RR, 2.42; 95% CI, 1.16-5.04) and 30 days (RR, 2.10; 95% CI, 1.06-4.16).

**Conclusion:**

Visual impairment was not associated with overall concussion incidence but was associated with approximately 2-fold higher risk of concussion coded with LOC. These findings suggest visual function may influence susceptibility to more clinically apparent concussive injuries and warrant consideration during preparticipation evaluation. More prospective studies are needed.

Mild traumatic brain injuries, commonly referred to as concussions, represent a significant public health concern in youth football. Prior studies estimate that approximately 3% to 5% of youth football players sustain a concussion during a single season, with higher rates observed in older age groups and competitive levels.^2,26^ Concussions in football most commonly result from player-to-player contact, particularly during tackling and running plays, which together account for the majority of reported injuries.^25^

Children and adolescents may be particularly vulnerable to the effects of concussion, as recovery trajectories can be prolonged compared with adults. Pediatric concussions have been associated with persistent symptoms and potential long-term sequelae, including academic difficulties, headaches, autonomic dysfunction, and neurocognitive impairments.^9,12,18^ Although the full long-term implications of repetitive head injury in youth athletes remain incompletely understood, identifying potentially modifiable risk factors is a critical component of injury prevention efforts in this population.^15,21^

Vision plays a pivotal role in athletic performance, especially in football, where players must accurately judge distances, detect peripheral movements, and react swiftly to dynamic play situations.^11^ Depth perception enables athletes to assess the spatial relationship between themselves, the ball, and other players, facilitating precise movements and strategic positioning.^17^ Peripheral vision allows players to be aware of their surroundings without direct focus, which is essential for monitoring opponents and teammates.^27^ Additionally, a quick visual reaction time is crucial for responding to rapid changes on the field, such as sudden passes or defensive maneuvers.^27^

Prior epidemiologic studies have characterized the incidence and mechanisms of concussion in youth football and other contact sports. Surveillance and cohort studies demonstrate that most concussions occur during tackling and running plays, with risk increasing alongside age and physical maturity.^13,20,23^ These investigations have primarily focused on external factors such as injury mechanism, player age, and exposure, as well as broader biomechanical contributors to head injury.^19,22,28^ To our knowledge, no large-scale population-based cohort study has evaluated clinically documented visual impairment as a predictor of concussion risk in youth football.

Complementary experimental and narrative studies have examined cognitive, perceptual, and sensory processes involved in concussion risk. Prior work has demonstrated that anticipation, reaction time, and sensory integration influence an athlete’s ability to prepare for and mitigate head impacts, suggesting a potential role for visual and perceptual function in injury susceptibility.^10,14^ However, this literature has largely been mechanistic in nature and has not evaluated whether clinically documented visual impairment is associated with concussion occurrence at the population level, particularly among youth football athletes. Systematic reviews of concussion risk factors have similarly highlighted the limited investigation of visual function as a predictor of injury in contact sports.^1^

Despite extensive research on concussion epidemiology and biomechanics, the role of clinically documented visual impairment as a potential risk factor in youth football remains poorly characterized. Addressing this gap is clinically relevant, as visual impairments are common in children and may be underrecognized during routine sports preparticipation evaluations.^29^ Improved understanding of the association between visual impairment and concussion risk could inform targeted screening strategies, individualized counseling, and injury prevention efforts.^24^ Therefore, the objective of this study was to evaluate the association between documented visual impairment and concussion occurrence among youth football athletes using a large national health care database. We hypothesized that youth athletes with documented visual impairment would have a higher incidence of concussion compared with those without visual impairment.

## Methods

A retrospective cohort analysis was conducted using the TriNetX US Collaborative Network, a federated health research platform that aggregates deidentified, longitudinal electronic health record data from approximately 70 health care organizations. The database is compliant with the Health Insurance Portability and Accountability Act, and the study was determined to constitute non–human subjects research by the University of California, Riverside, School of Medicine. Study cohorts, covariates, and outcomes were identified using the International Classification of Diseases, Tenth Revision (ICD-10) coding system.

**Figure 1.**
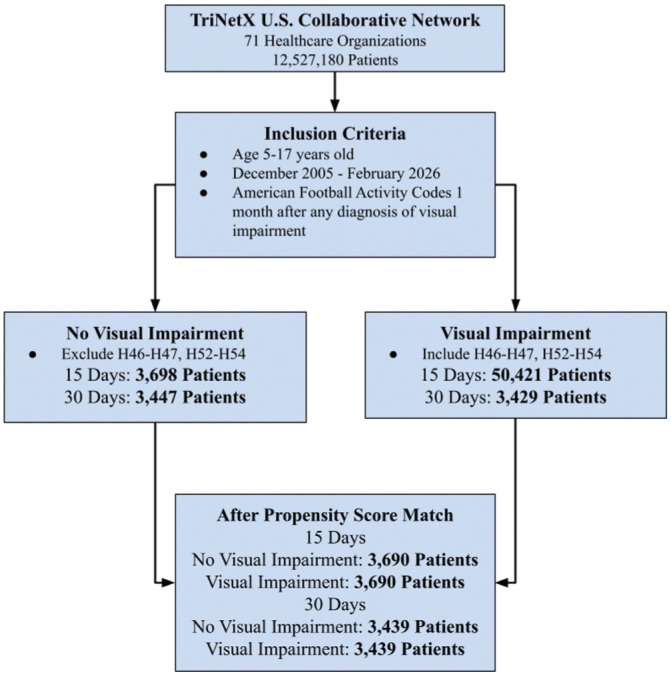
Flow diagram illustrating cohort identification within the TriNetX US Collaborative Network. Pediatric patients aged 5 to 17 years with documented football-related ICD-10 activity codes between December 2005 and February 2026 were identified. Patients were stratified based on the presence or absence of visual impairment defined by ICD-10 codes H46 to H47 and H52 to H54, with visual impairment diagnoses required to precede the index football encounter by ≥1 month. Cohort sizes before and after 1:1 propensity score matching are shown for 15- and 30-day outcome windows.

**Figure 2.**
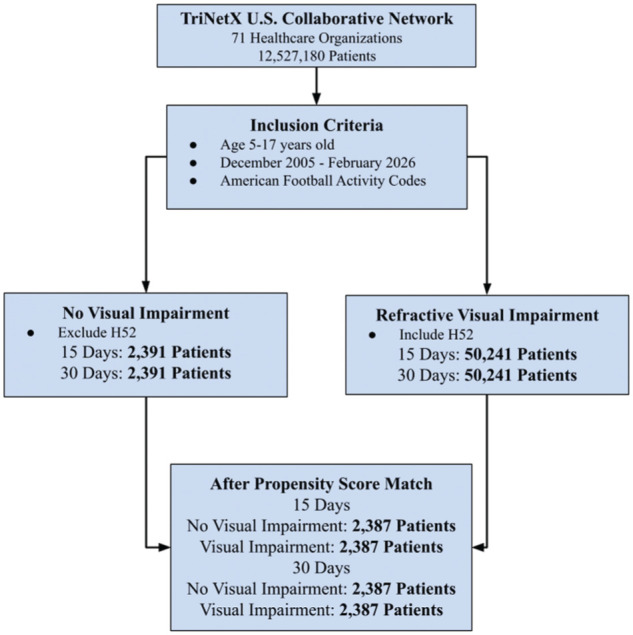
Flow diagram illustrating cohort identification within the TriNetX US Collaborative Network. Pediatric patients aged 5 to 17 years with documented football-related ICD-10 activity codes between December 2005 and February 2026 were identified. Patients were stratified based on the presence or absence of refractive visual impairment defined by ICD-10 codes H52 with visual impairment diagnoses required to precede the index football encounter by ≥1 month. Cohort sizes before and after 1:1 propensity score matching are shown for 15- and 30-day outcome windows.

A total of 52,801 pediatric athletes aged 5 to 17 years who played football and were documented in the TriNetX national database were identified. Data were queried February 2026 and included data from December 2005 to February 2026. Among them, 3680 athletes had visual impairments and 44,638 did not. The index encounter was defined as the first recorded football-related ICD-10 code during the study period for each patient, and outcomes were assessed within 15- and 30-day windows following the index encounter to evaluate incident concussion diagnoses temporally associated with football participation. Football participation was defined by the presence of ≥1 ICD-10 code indicating football activity or football-related injury (Y93.61, W21.81XA, W21.01XA). While ICD-10 activity and external cause codes may not capture all instances of football participation, this approach has been used in prior administrative database studies examining sports-related injury patterns.^9^ General visual impairment was defined using ICD-10 codes H46 to H47, H52, and H53 to H54, which were grouped together to increase statistical power. Given variability in documentation of visual acuity within administrative data sets, ICD-10 categories were used to identify clinically documented visual disorders rather than specific Snellen acuity thresholds. Diagnosis of a visual deficiency must have been made 1 month before the football encounter. Concussions were broadly determined using the S06.0X0 to S06.0X9 code (Figure 1). To address heterogeneity within visual impairment as defined by ICD-10, we performed stratified analyses looking at just refractive disorders (H52); thus, the refractive disorder group was defined as patients aged 5 to 17 years with a football code and the presence of a refractive disorder (Figure 2). For our analysis specific to loss of consciousness (LOC), we used ICD-10 codes S06.0X1 to S06.0X9. Only concussion diagnoses occurring after the index encounter were included as outcomes.

Patient demographics were collected including age, sex, race, and ethnicity. Primary outcomes were the incidence of concussion and concussion with LOC. All statistical analyses were performed using TriNetX platform, including calculation of incidence proportions, risk ratios (RRs) with 95% CIs, *P* values using 2-sided tests, and 1:1 propensity score matching with assessment of covariate balance using standardized mean differences (SMDs). A full list of codes used in this study can be found in Table 1. Propensity score matching was conducted using a 1:1 greedy nearest-neighbor algorithm based on age, sex, body mass index, anxiety, depression, prior concussion diagnosis recorded before the index encounter, and intellectual disability, with covariate balance assessed by SMD < 0.1 considered acceptable (Table 2).

**Table 1:** Full List of Codes Used for Cohort Creation, PSM, and Outcome Analysis^*a*^

Domain	ICD-10 Code(s)	Variable Included
Football activity codes	Y93.61	Activity, American tackle football
	W21.81XA	Striking against or struck by football helmet, initial encounter
	W21.01XA	Struck by football, initial encounter
PSM codes		
Demographics	AI	Age at index (continuous)
	M,F	Sex (male, female)
	2106-3	White
	2054-5	Black or African American
Neurodevelopmental disorders	F90.2	ADHD, combined type
	F81	Specific developmental disorders of scholastic skills
Psychiatric disorders	F33	Major depressive disorder, recurrent
	F41	Other anxiety disorders
Headache disorders	G43	Migraine
	G44	Other headache syndromes
Metabolic disorders	E08-E13	Diabetes mellitus
Social determinants of health	Z55-Z65	Socioeconomic and psychosocial risk factors
Prior concussion	S06.0	History of concussion prior to index
BMI	TNX curated	BMI (continuous and categorical: <18, 18-24.99, 25-29.99, 30-34.99, 35-39.99, 40-45 kg/m^2^)
Outcome codes		
Concussion	S06.0	Concussion
Concussion with LOC	S06.0X1	Concussion with LOC of 30 minutes or less
	S06.0X2	Concussion with LOC of 31 to 59 minutes
	S06.0X3	Concussion with LOC of 1 hour to 5 hours 59 minutes
	S06.0X4	Concussion with LOC of 6 hours to 24 hours
	S06.0X9	Concussion with LOC of unspecified duration

a ADHD, attention-deficit hyperactivity disorder; BMI, body mass index; ICD-10, International Classification of Diseases, Tenth Revision; LOC, loss of consciousness; TNX, TriNetX.

**Table 2:** Baseline Characteristics and Covariate Balance Before and After PSM^*a*^

Covariate (ICD-10 Code)	Analysis	Pre-PSM Value	Pre-PSM SMD	Post-PSM Value	Post-PSM SMD
AI — age at index	General VI vs no general VI	3698 vs 50241	0.07	3690 vs 3690	0.01
	Refractive impairment vs no refractive impairment	2391 vs 50241	0.01	2387 vs 2387	0.01
M — male	General VI vs no general VI	3398 vs 47103	0.07	3393 vs 3407	0.01
	Refractive impairment vs no refractive impairment	2209 vs 47103	0.05	2207 vs 2217	0.02
2106-3 — White	General VI vs no general VI	1942 vs 27663	0.05	1937 vs 1961	0.01
	Refractive impairment vs no refractive impairment	1276 vs 27663	0.03	1274 vs 1306	0.03
2054-5 — Black or African American	General VI vs no general VI	1133 vs 13655	0.08	1131 vs 1135	0.002
	Refractive impairment vs no refractive impairment	713 vs 13655	0.06	712 vs 706	0.01
F — female	General VI vs no general VI	300 vs 3134	0.07	297 vs 283	0.01
	Refractive impairment vs no refractive impairment	182 vs 3134	0.05	180 vs 170	0.02
Z55-Z65 — persons with potential health hazards related to socioeconomic and psychosocial circumstances	General VI vs no general VI	464 vs 1793	0.33	459 vs 490	0.03
	Refractive impairment vs no refractive impairment	309 vs 1793	0.35	306 vs 328	0.03
F41 — other anxiety disorders	General VI vs no general VI	436 vs 1852	0.31	430 vs 424	0.01
	Refractive impairment vs no refractive impairment	274 vs 1852	0.30	270 vs 286	0.02
S06.0 — concussion	General VI vs no general VI	401 vs 1939	0.27	396 vs 403	0.01
	Refractive impairment vs no refractive impairment	181 vs 1939	0.16	179 vs 175	0.01
F90.2 — attention-deficit hyperactivity disorder, combined type	General VI vs no general VI	379 vs 1863	0.26	376 vs 414	0.03
	Refractive impairment vs no refractive impairment	244 vs 1863	0.26	243 vs 237	0.01
G44 — other headache syndromes	General VI vs no general VI	316 vs 1009	0.30	310 vs 295	0.01
	Refractive impairment vs no refractive impairment	148 vs 1009	0.21	147 vs 148	0.002
G43 — migraine	General VI vs no general VI	240 vs 807	0.25	235 vs 215	0.02
	Refractive impairment vs no refractive impairment	132 vs 807	0.21	132 vs 118	0.03
F81 — specific developmental disorders of scholastic skills	General VI vs no general VI	225 vs 633	0.26	222 vs 212	0.01
	Refractive impairment vs no refractive impairment	155 vs 633	0.27	151 vs 134	0.03
E08-E13 — diabetes mellitus	General VI vs no general VI	52 vs 186	0.11	51 vs 43	0.02
	Refractive impairment vs no refractive impairment	33 vs 186	0.11	31 vs 29	0.01
F33 — major depressive disorder, recurrent	General VI vs no general VI	28 vs 116	0.08	27 vs 17	0.04
	Refractive impairment vs no refractive impairment	16 vs 116	0.07	16 vs 16	0
9083 — BMI	General VI vs no general VI	2275 vs 17839	0.06	2267 vs 2069	0.04
	Refractive impairment vs no refractive impairment	1519 vs 17839	0.01	1515 vs 1326	0.10
9083 — BMI (<18 kg/m^2^)	General VI vs no general VI	1823 vs 13488	0.48	1815 vs 1831	0.01
	Refractive impairment vs no refractive impairment	1178 vs 13488	0.47	1174 vs 1178	0.003
9083 — BMI (18-24.99 kg/m^2^)	General VI vs no general VI	1525 vs 10417	0.45	1519 vs 1574	0.03
	Refractive impairment vs no refractive impairment	993 vs 10417	0.46	989 vs 1011	0.02
9083 — BMI (25-29.99 kg/m^2^)	General VI vs no general VI	577 vs 3834	0.25	574 vs 563	0.01
	Refractive impairment vs no refractive impairment	351 vs 3834	0.23	349 vs 348	0.001
9083 — BMI (30-34.99 kg/m^2^)	General VI vs no general VI	309 vs 1950	0.19	306 vs 286	0.02
	Refractive impairment vs no refractive impairment	183 vs 1950	0.16	182 vs 173	0.01
9083 — BMI (35-39.99 kg/m^2^)	General VI vs no general VI	139 vs 806	0.13	136 vs 101	0.05
	Refractive impairment vs no refractive impairment	80 vs 806	0.11	80 vs 61	0.05
9083 — BMI (40-45 kg/m^2^)	General VI vs no general VI	64 vs 279	0.11	63 vs 40	0.05
	Refractive impairment vs no refractive impairment	37 vs 279	0.10	37 vs 23	0.05

a Codes used to define football participation, propensity score matching PSM variables, and concussion outcomes in the TriNetX database. Demographic variables were identified using structured electronic health record data. BMI was obtained from TriNetX-curated fields. Prior concussion was defined as any S06.0 diagnosis occurring before the index encounter. BMI, body mass index; ICD-10, International Classification of Diseases, Tenth Revision; PSM, propensity score matching; SMD, standardized mean difference; VI, visual impairment.

## Results

A total of 52,801 pediatric athletes aged 5 to 17 years with documented football participation were identified within the TriNetX national database. Of these, 3680 had a recorded diagnosis of general visual impairment and 44,638 had no documented visual impairment. After 1:1 propensity score matching, 3674 athletes with general visual impairment were matched to 3674 athletes without visual impairment. In the refractive-only subgroup analysis, 2390 matched patients were included.

The matched cohort represented a demographically diverse population with substantial representation across racial and ethnic groups. Following propensity score matching, baseline demographic and clinical characteristics were well balanced between exposure groups (SMDs < 0.1 for all covariates) (Table 1).

### General Visual Impairment

Within 15 days of the index football-related encounter, the incidence of concussion was 1.65% in the general visual impairment group compared with 1.47% in matched controls (RR, 1.12; 95% CI, 0.75-1.68; *P* = .57). At 30 days, concussion incidence was 1.79% in the visual impairment group and 1.36% in controls (RR, 1.09; 95% CI, 0.74-1.59; *P* = .67), demonstrating no statistically significant difference (Table 3).

**Table 3:** Incidence of Concussion in Children With General VI and No VI^*a*^

Time	Cohort	Concussion, n	RR (95% CI)	RD, % (95% CI)	*P*
0-15 d	No impairment	44	1.123 (0.751 to 1.679)	0.181 (–0.445 to 0.808)	.57
	General VI	50			
0-30 d	No impairment	50	1.087 (0.744 to 1.589)	0.146 (–0.516 to 0.807)	.67
	General VI	55			

a RRs and RDs are shown with 95% CIs comparing athletes with general VI to those without VI after 1:1 propensity score matching. RD, risk difference; RR, risk ratio; VI, visual impairment.

In contrast, concussion with LOC was significantly more frequent among visually impaired athletes. At 15 days, 36 concussions with LOC occurred in the visual impairment group compared with 17 in controls (RR, 2.12; 95% CI, 1.19-3.76; *P* = .009). At 30 days, the association persisted (RR, 2.11; 95% CI, 1.21-3.69; *P* = .007), with a corresponding risk difference of approximately 0.57% (Table 4).

**Table 4:** Incidence of Concussion With LOC in Children With General VI and No VI^*a*^

Time	Cohort	Concussion With LOC, n	RR (95% CI)	RD, % (95% CI)	*P*
0-15 d	No impairment	17	2.117 (1.191,3.762)	0.537 (0.135,0.939)	.**009**
	General VI	36			
0-30 d	No impairment	18	2.111 (1.207,3.691)	0.565 (0.152,0.978)	.**007**
	General VI	38			

a Bold indicates significant data (*P* < .05). RRs and RDs with 95% CIs compare athletes with general VI to those without VI following 1:1 propensity score matching. LOC, loss of consciousness; RD, risk difference; RR, risk ratio; VI, visual impairment.

### Refractive Visual Impairment Subgroup

**Figure 3.**
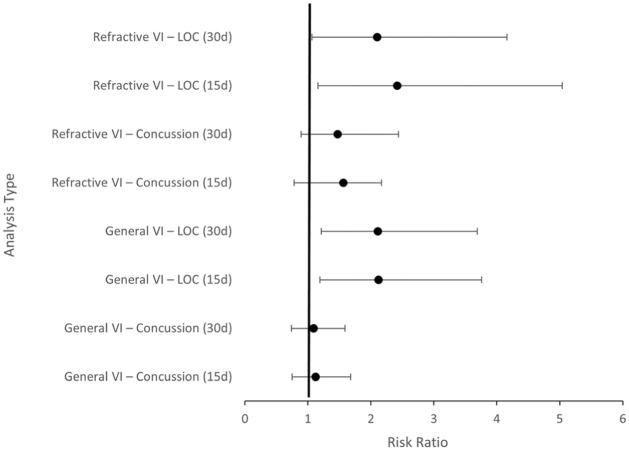
Risk ratios (RRs) and 95% CIs are shown for overall concussion and concussion with loss of consciousness (LOC) at 15 and 30 days following the index football-related encounter. The vertical line represents RR = 1. VI, visual impairment. Asterisks indicate statistical significance (*P* < .05).

Among athletes with refractive visual impairment, overall concussion incidence was not significantly different from controls at either 15 days (RR, 1.56; 95% CI, 0.78-2.12; *P* = .33) or 30 days (RR, 1.47; 95% CI, 0.89-2.44; *P* = .13) (Table 5). However, concussion with LOC was significantly associated with refractive visual impairment. At 15 days, the RR was 2.42 (95% CI, 1.16-5.04; *P* = .02). At 30 days, the association remained statistically significant (RR, 2.10; 95% CI, 1.06-4.16; *P* = .03) (Table 6 and Figure 3).

**Table 5:** Incidence of Concussion in Children With Refractive VI and No VI^*a*^

Time	Cohort	Concussion, n	RR (95% CI)	RD, % (95% CI)	*P*
0-15 d	No impairment	25	1.564 (0.7773 to 2.169)	0.368 (–0.364 to 1.100)	.33
	Refractive VI	33			
0-30 d	No impairment	25	1.473 (0.89 to 2.438)	0.601 (–0.174 to 1.376)	.13
	Refractive VI	37			

a RRs and RDs with 95% CIs compare athletes with refractive VI to those without VI following 1:1 propensity score matching. RD, risk difference; RR, risk ratio; VI, visual impairment.

**Table 6:** Incidence of Concussion With LOC in Children With Refractive VI and No Refractive VI^*a*^

Time	Cohort	Concussion With LOC, n	RR (95% CI)	RD, % (95% CI)	*P*
0-15 d	No impairment	10	2.415 (1.157-5.038)	0.611 (0.117-1.105)	.**02**
	Refractive VI	24			
0-30 d	No impairment	12	2.096 (1.056-4.162)	0.568 (0.053-1.083)	.**03**
	Refractive VI	25			

a Bold indicates significant data (*P* < .05) RRs and RDs with 95% CIs compare athletes with refractive VI to those without refractive VI following 1:1 propensity score matching. RD, risk difference; RR, risk ratio; VI, visual impairment. LOC, loss of consciousness.

## Discussion

In this large, propensity score–matched cohort of youth football athletes, documented visual impairment was not consistently associated with overall concussion incidence within 15- and 30-day windows following a football-related encounter. However, visual impairment was significantly associated with concussion coded with LOC. This association was observed in both the broader visual impairment cohort and the refractive visual impairment subgroup, with approximately 2-fold higher relative risk compared with matched athletes without documented visual impairment. These findings suggest that while visual impairment may not be associated with overall concussion diagnoses in administrative data, it may be associated with an increased likelihood of more clinically apparent concussive injuries.

The observed association with concussion accompanied by LOC may reflect underlying visual and sensory factors relevant to collision sports. Effective anticipation of contact in football relies on intact visual acuity, peripheral awareness, depth perception, and visual-motor integration.^21^ Athletes with impaired visual function may have diminished capacity to detect and prepare for incoming impacts, potentially reducing their ability to brace or adjust body positioning prior to collision. Prior mechanistic studies have demonstrated that anticipation and visual processing influence head acceleration and injury susceptibility during contact events.^16,17^ While causal mechanisms cannot be established from administrative data, the present findings are consistent with the hypothesis that compromised visual function may influence vulnerability to certain types of concussive injury, especially those leading to LOC.

Studies have demonstrated that visually impaired athletes may be at higher risk for sports-related concussions. Although a different population, an English Para Athletics study among athletes with vision impairment reported a significantly higher incidence of sport-related concussion compared with their counterparts with normal vision, with collisions being the most common injury mechanism.^27^ This suggests that visual impairment could be a significant risk factor for concussions in contact sports like football. The importance of visual function in concussion risk is further highlighted by research on eye discipline in soccer players. Clark et al^5^ observed that female soccer players were more likely to close their eyes while heading a ball compared with male players, potentially increasing their concussion risk due to reduced awareness of the ball's velocity and inability to anticipate impact intensity. While the Clark et al^5^ study focused on soccer, similar principles could apply to football, where visual awareness is crucial for avoiding or preparing for collisions.

Although visual impairment was associated with concussion coded with LOC, the relationship with overall concussion diagnoses was less consistent across time intervals. Several factors may explain this finding. First, mild concussions without LOC may be underdiagnosed or inconsistently documented in administrative data sets, particularly if symptoms are transient or do not prompt medical evaluation.^12^ In contrast, concussions accompanied by LOC are more likely to result in acute clinical assessment and formal coding, leading to more reliable capture within electronic health records. Second, the modest risk differences observed in this study may limit statistical power to detect smaller effect sizes for overall concussion incidence. Finally, concussion is a heterogeneous clinical entity with variable presentation, and administrative coding may not fully capture differences in symptom burden or injury severity.^6^ Together, these factors may contribute to the attenuation of association observed for overall concussion outcomes while a more consistent signal was detected for concussion with LOC.

Although the risk differences observed in this study were modest, the relative increase in concussion coded with LOC warrants consideration. Even small increases in risk may translate into a meaningful number of additional injuries at the population level given the high participation rates in youth football nationwide and potential limitations in coding leading to decreased sample sizes in this study. Importantly, visual impairment represents an identifiable clinical characteristic that is routinely encountered in pediatric practice. While causality cannot be inferred from this observational analysis, these findings suggest that clinicians may consider incorporating more deliberate assessment of visual function during preparticipation evaluations and ensuring that documented visual impairments are optimally corrected prior to contact sport participation. Individualized counseling for athletes with known visual deficits may represent a pragmatic approach to risk awareness without restricting participation. Further prospective investigation is needed to determine whether targeted visual assessment or optimization meaningfully alters injury risk.

These findings carry important implications for clinicians, families, coaches, and athletes. First, sports physicals and preparticipation evaluations for youth athletes should include routine visual acuity screenings. Importantly, visual impairment represents a potentially identifiable and modifiable characteristic. Enhanced attention to vision assessment, optimization of corrective lenses, and targeted counseling for athletes with known visual deficits may represent practical strategies to mitigate risk. These findings support consideration of visual function as one component of comprehensive preparticipation evaluation and injury prevention efforts.

Currently, the Snellen test is the preferred test for routine sports physicians, with a single eye greater than 20/40 vision requiring a follow-up with an eye specialist to receive clearance. While the Snellen test provides convenience for physicians, it has the potential to miss disorders that may not fully capture aspects of functional vision potentially relevant to collision sports.^16^ Additionally, previous studies have noted that standard Snellen distance testing may not be sufficient to identify certain visual deficiencies. These findings raise questions about whether standard distance acuity testing fully captures functional visual performance relevant to collision sports. Athletes with previously documented visual acuity disorders could receive further help via a referral to pediatric ophthalmology, to optimize visual input and mitigate the risk of injury.^12^

Second, sports medicine physicians and primary care providers must be more proactive in guiding visually impaired youth and their families on the risks associated with contact sports. In some cases, this may involve engaging in shared decision-making regarding sport participation, seeking appropriate visual correction, or changing positions to one with a lower collision risk. In sports, some positions have an inherently higher risk of concussion than others.^4,7^ Sports medicine physicians must be knowledgeable about the risks of injury in sports so that they can provide further guidance to their athletes. This personalized approach to injury prevention can help balance the physical and social benefits of sports participation with the safety needs of vulnerable athletes.

Last, physicians should remain cognizant of cultural and socioeconomic factors that may influence a family's decision-making process around protective equipment. In some communities, the stigma of wearing sports goggles or additional padding may deter athletes from adopting recommended protective gear.^8^ A 2001 study noted that vision problems were noted in a high percentage of athletes, yet many chose not to wear protective and corrective eyewear.^3^ Understanding these attitudes through culturally sensitive dialogue can help clinicians better counsel patients and promote safer behaviors in a way that respects family and community values.

### Limitations

Several limitations should be considered when interpreting these findings. First, this was a retrospective analysis of administrative electronic health record data and therefore lacked granular clinical detail. Information regarding player position, mechanism of injury, exposure volume, and use of protective equipment was not available. These factors may influence concussion risk and could not be accounted for in the present study. Second, both visual impairment and concussion outcomes were defined using ICD-10 diagnostic codes. Administrative coding may incompletely capture true incidence due to variability in documentation practices, delayed care seeking, or underreporting.^9^ Third, the database did not reliably capture corrective lens use at the time of injury. Additionally, the number of athletes with documented visual impairment was relatively small compared with the overall football cohort, which may limit statistical power in subgroup analyses. The broad ICD-10 groupings used to define visual impairment may also obscure meaningful differences in severity or etiology, underscoring the need for more granular coding and prospective characterization of visual function in athletic populations.

Future research should incorporate prospective study designs with detailed, standardized assessments of visual function, including quantitative visual acuity measurements, depth perception, peripheral visual field testing, and visual-motor integration. Such studies would allow stratification by severity and type of visual impairment, rather than reliance on broad diagnostic codes, and could better clarify dose-response relationships between visual deficits and concussion risk. Additionally, integration of exposure metrics (eg, position played, snap counts, practice vs game exposure) and biomechanical data would strengthen causal inference. Future investigations should also be prospective in nature and track study participants with visual deficiencies throughout the course of a football season, seeing if they suffer concussions at higher rates than their peers. Finally, extending this work to other collision and contact sports may help determine whether the observed association is unique to youth football or reflects a broader relationship between visual function and concussion susceptibility across athletic populations.

## Conclusion

In this propensity score–matched cohort of youth football athletes, visual impairment was associated with an increased risk of concussion coded with LOC, while associations with overall concussion diagnoses were less consistent. These findings suggest that visual function may play a role in susceptibility to more clinically apparent concussive injuries in contact sports. Although the risk differences were modest, even small increases in injury risk may have meaningful implications at the population level given the high participation rates in youth football. Visual impairment represents an identifiable characteristic that may warrant consideration during preparticipation evaluations. Further prospective studies incorporating detailed measures of visual acuity, functional vision, corrective lens use, injury mechanism, and exposure metrics are needed to better define the relationship between visual function and concussion risk and to inform evidence-based prevention strategies.

## References

[1] Abrahams S Fie SM Patricios J Posthumus M September AV . Risk factors for sports concussion: an evidence-based systematic review. Br J Sports Med. 2014;48(2):91-97. doi:10.1136/bjsports-2013-092734 24052371

[2] American Medical Society for Sports Medicine. Clear eyes, full hearts, cleared to play? Published September 18, 2014. Accessed January 10, 2026. https://www.amssm.org/clear_eyes_full_hearts_cle-csa-712.html

[3] Beckerman SA Hitzeman S . The ocular and visual characteristics of an athletic population. Optometry. 2001;72(8):498-509. 11519712

[4] Chatha K Pruis T Peaguda CF , et al. Concussions in soccer: an epidemiological analysis in the pediatric population. Orthop J Sports Med. 2020;8(10):2325967120951077. doi:10.1177/2325967120951077 PMC7588758 33173798

[5] Clark JF . Lack of eye discipline during headers in high school girls soccer: a possible mechanism for increased concussion rates. Med Hypotheses. 2017;100:10-14. doi:10.1016/j.mehy.2016.12.016 28236839

[6] Corwin DJ Arbogast KB Haber RA , et al. Characteristics and outcomes for delayed diagnosis of concussion in pediatric patients presenting to the emergency department. J Emerg Med. 2020;59(6):795-804. doi:10.1016/j.jemermed.2020.09.017 33036827 PMC7736137

[7] Dai JB Li AY Haider SF , et al. Effects of game characteristics and player positions on concussion incidence and severity in professional football. Orthop J Sports Med. 2018;6(12):2325967118815448. doi:10.1177/2325967118815448 PMC6311573 30627588

[8] Dean NA Bundon A . “Helmets aren’t cool”: surfers’ perceptions and attitudes towards protective headgear. Int Rev Sociol Sport. 2020;56(5):739-756. doi:10.1177/1012690220931736

[9] Finch CF Boufous S . Do inadequacies in ICD-10-AM activity coded data lead to underestimates of the population frequency of sports/leisure injuries?. Inj Prev. 2008;14(3):202-204. doi:10.1136/ip.2007.017251 18523115

[10] Haarbauer-Krupa J Lee AH Bitsko RH Zhang X Kresnow-Sedacca MJ . Prevalence of parent-reported traumatic brain injury in children and associated health conditions. JAMA Pediatr. 2018;172(11):1078-1086. doi:10.1001/jamapediatrics.2018.2740 30264150 PMC6248161

[11] Hallock H Mantwill M Vajkoczy P , et al. Sport-related concussion: a cognitive perspective. Neurol Clin Pract. 2023;13(2):e200123. doi:10.1212/CPJ.0000000000200123 PMC9987206 36891462

[12] Kamil R Atef AbdelAlim Y Patel S , et al. Objective markers for diagnosing concussions: beyond blood biomarkers and the role of real-time diagnostic tools. J Clin Med. 2025;14(21):7727. doi:10.3390/jcm14217727 41227122 PMC12609333

[13] Kung SM Suksreephaisan TK Perry B Palmer BR Page RA . The effects of anticipation and visual and sensory performance on concussion risk in sport: a review. Sports Med Open. 2020;6:54. doi:10.1186/s40798-020-00283-6 33196878 PMC7669979

[14] Kurowski BG Haarbauer-Krupa J Giza CC . When traumatic brain injuries in children become chronic health conditions. J Head Trauma Rehabil. 2023;38(4):348-350. doi:10.1097/HTR.0000000000000842 36584980 PMC10310882

[15] LaBella C . Youth tackle football: perception and reality. Pediatrics. 2019;143(5):e20190519. doi:10.1542/peds.2019-0519 30936252

[16] Langlois JA Rutland-Brown W Wald MM . The epidemiology and impact of traumatic brain injury: a brief overview. J Head Trauma Rehabil. 2006;21(5):375-378. doi:10.1097/00001199-200609000-00001 16983222

[17] Lochhead L Feng J Laby DM Appelbaum LG . Training vision in athletes to improve sports performance: a systematic review of the literature. Int Rev Sport Exerc Psychology. Published online December 9, 2024. doi:10.1080/1750984x.2024.2437385

[18] Manzanero S Elkington LJ Praet SF Lovell G Waddington G Hughes DC . Post-concussion recovery in children and adolescents: a narrative review. Concussion. 2017;1:2059700217726874. doi:10.1177/2059700217726874

[19] Meaney DF Smith DH . Biomechanics of concussion. Clin Sports Med. 2011;30(1):19-31. doi:10.1016/j.csm.2010.08.009 21074079 PMC3979340

[20] Pfister T Pfister K Hagel B Ghali WA Ronksley PE . The incidence of concussion in youth sports: a systematic review and meta-analysis. Br J Sports Med. 2016;50(5):292-297. doi:10.1136/bjsports-2015-094978 26626271

[21] Reuter-Rice K Fitterer AN Duquette P , et al. A study protocol for risk stratification in children with concussion (RSiCC): Theoretical framework, design, and methods. PLoS One. 2024;19(7):e0306399. Published online July 18, 2024. doi:10.1371/journal.pone.0306399 PMC11257289 39024215

[22] Rowson S Bland ML Campolettano ET , et al. Biomechanical perspectives on concussion in sport. Sports Med Arthrosc Rev. 2016;24(3):100-107. doi:10.1097/JSA.0000000000000121 27482775 PMC4975525

[23] Schallmo MS Weiner JA Hsu WK . Sport and sex-specific reporting trends in the epidemiology of concussions sustained by high school athletes. J Bone Joint Surg Am. 2017;99(15):1314-1320. doi:10.2106/JBJS.16.01573 28763417

[24] Swanson MW Weise KK Dreer LE , et al. Academic difficulty and vision symptoms in children with concussion. Optom Vis Sci. 2017;94(1):60-67. doi:10.1097/OPX.0000000000000977 27668641 PMC5182095

[25] Theofilou G Ladakis I Mavroidi C , et al. The effects of a visual stimuli training program on reaction time, cognitive function, and fitness in young soccer players. Sensors. 2022;22(17):6680. doi:10.3390/s22176680 36081136 PMC9460176

[26] U.S. Centers for Disease Control and Prevention (CDC) Heads Up. Data on sports and recreation activities. August 26, 2024. Accessed January 10, 2026. https://www.cdc.gov/heads-up/data/index.html

[27] Vater C Wolfe B Rosenholtz R . Peripheral vision in real-world tasks: a systematic review. Psychon Bull Rev. 2022;29(5):1531-1557. doi:10.3758/s13423-022-02117-w 35581490 PMC9568462

[28] Weiler R Ahmed OH Mechelen WV Verhagen E Bolling C . Concussion through my eyes: a qualitative study exploring concussion experiences and perceptions of male English blind footballers. Br J Sports Med. 2023;57(10):578-589. doi:10.1136/bjsports-2022-106256 36792363

[29] Weise KK Galt SJ Hale MH Springer DB Swanson MW . Pre-participation vision screening and comprehensive eye care in National Collegiate Athletic Association athletes. Optom Vis Sci. 2021;98(7):764-770. doi:10.1097/OPX.0000000000001738 34328455

